# High risk of virologic failure among HIV-infected children and adolescents routinely followed-up in Littoral region of Cameroon

**DOI:** 10.1371/journal.pone.0289426

**Published:** 2023-08-10

**Authors:** Patient Juste Mbébi Enoné, Calixte Ida Penda, Grâce Ngondi, Joseph Fokam, Serge Bruno Ebong, Jerson Mekoulou Ndongo, Estelle Géraldine Essangui Same, Louis Sides Ndjengue Nson, Samuel Honoré Mandengue, Carole Else Eboumbou Moukoko

**Affiliations:** 1 Faculty of Sciences, Department of Animal Biology and Physiology, University of Douala, Douala, Cameroon; 2 Faculty of Medicine and Pharmaceutical Sciences, Clinical Sciences Department, University of Douala, Douala, Cameroon; 3 Accredited HIV Care Center, Laquintinie Hospital of Douala, Douala, Cameroon; 4 Faculty of Medicine and Pharmaceutical Sciences, Biological Sciences Department, University of Douala, Douala, Cameroon; 5 Biological Laboratory, Laquintinie Hospital of Douala, Douala, Cameroon; 6 Chantal BIYA International Reference Centre for Research on HIV/AIDS Prevention and Management (CIRCB), Yaounde, Cameroon; 7 Faculty of Health Sciences, Department of Medical Laboratory Science, University of Buea, Buea, Cameroon; 8 Laboratory of Parasitology, Mycology and Virology, Postgraduate Training Unit for Health Sciences, Postgraduate School for Pure and Applied Sciences, University of Douala, Douala, Cameroon; 9 Centre Pasteur of Cameroon, Yaounde, Cameroon; Nigerian Institute of Medical Research, NIGERIA

## Abstract

Virological response to antiretroviral therapy (ART) remains a challenge for HIV-infected children and adolescents due to non-optimization of pediatric ART for resource-limited settings. In this study, we aimed to investigate factors associated with virologic failure (VF) in HIV-infected-children and adolescents on ART in Cameroon. A prospective patient-based cohort study was conducted among HIV-infected children (0–9 years) and adolescents (10–19 years) followed-up between November 2018 and October 2019 in 38 healthcare centers located in the Littoral region of Cameroon. The 1^st^ viral load (VL) was assessed after 6 months of ART initiation and the 2^nd^ VL between 3 and 6 six months later in patients with VL ≥1000 copies/ml in accordance with the national algorithm using Abbott Real-Time HIV-1 Viral Load Assay. Multivariate analyses were performed to identify the determinants of higher risk of VF. Of 1,029 HIV-infected children and adolescents (393 children and 636 adolescents), 801 (77.8%) cumulatively presented with VL <1000 copies/mL within 12 months on ART. Adolescents were more likely to have VF than children (24.5% *vs* 18.3%, OR: 1.39; 95%CI: 1.00–1.93; p = 0.047). Patients followed-up in decentralized care units were significantly more likely to have VF compared to those attending the accredited treatment centers (26.1% *vs* 16.6%, OR: 1.88, 95%CI: 1.37–2.58; p<0.001). Our findings show a high rate of VL suppression (VLS, 77.8%) among HIV-infected children and adolescents, albeit lower than the established target of 90%. Being adolescent and patients followed in the decentralized care units are high risk factors for VF, thereby necessitating routine therapeutic education of patients and guardians in resource limited countries to improve VLS.

## Introduction

Although global incidence and mortality due to Human Immunodeficiency virus (HIV) have declined, HIV infection remains a major public health problem in several parts of the world. In 2018, approximately 37.9 million people were living with HIV worldwide, including 1.7 million children and adolescents aged 0 to 14 years, of whom 160,000 were new infections [1–3]. Sub-Saharan Africa remains the most affected region with more than 80% of HIV-infected children and young adolescents worldwide [2]. In West and Central Africa, approximately 440,000 HIV-infected children and young adolescents were reported, of whom 58,000 were new infections, and 39,000 deaths [3]. Since 2016, the United Nations has set a major goal of ending the HIV pandemic by 2030, by reducing HIV incidence and providing antiretroviral treatment (ART) to infected people [4]. In 2018, only 54% of HIV-infected children and adolescents had access to ART globally and 28% in West and Central Africa [2,3]. It is therefore imperative to emphasize on the follow-up of HIV-infected children and adolescents in order to improve the actions taken and the virological and clinical results in this population.

Good adherence to ART is a key determinant of viral load suppression (VLS), but it is rarely observed in most patients with chronic disease, including in HIV-infected patients [5,6]. Treatment compliance in children and adolescents is complex, and it is influenced by a variety of factors, including drug regimen, patient and family factors, and relationship with healthcare personnel [7–10]. Some studies have shown that HIV-infected children and adolescents are less likely to adhere to ART and remain in the care system. Indeed, children and adolescents may have great difficulty adhering to ART when initiated at early childhood, and are more likely than adults to experience treatment failure and emergence of HIV drug resistance (HIVDR) [11].

Unfortunately, in Central Africa, there is limited data on VLS or virologic failure (VF) in the HIV-infected children and adolescents. Most studies do not provide information on VLS at specific time point, and VLS are highly variable in this population [12]. Previous studies in East and West Africa have reported alarming rates of VF among HIV-infected adolescents, ranging from 33.0% in Zimbabwe to 51.6% in Togo, and these VF were primarily associated with a high prevalence of drug resistance mutations [13,14].

Cameroon is located in Central Africa and adheres to the three strategic objectives of the UNAIDSs: 90% of HIV-infected people will know their serological status, 90% of HIV-infected people knowing their status receive sustained ART, and 90% of patients on ART have VLS by 2020 [15,16]. Recently, this target was increased to 95% by 2025 [17]. Achieving these goals remains a challenge and requires several approaches such as improving access to optimized treatment, availability of ART and integrating innovative strategies including the implementation of “test and treat" [17]. In 2018, about 43,000 of the HIV-infected children and adolescents were reported in Cameroon leading to 3600 deaths; 4,500 were new infections and 24% had access to ART [18]. Since the implementation of the “test and treat" strategy in Cameroon in 2016, four published studies with sample sizes ranging from 71 to 190 HIV-infected children and/or adolescents have evaluated virological responses to ART [19–22]. In the studies conducted in Central, Southern and Eastern regions of Cameroon, the VLS rate varied from 53.3% to 79.31% in adolescents and from 74.7% to 80.0% in children [19,21,22]. Two recent studies carried out in Douala among adolescents aged 10–19 years and HIV-infected individuals aged 15–64 years have reported VF rates of 16.3% and 39.2%, respectively, and depended on the healthcare facilities [23,24]. Sociodemographic, clinical and treatment characteristics have also been associated with poor VLS in Cameroon [19–22] and elsewhere [25–30]. Here, we investigated the prevalence of VF among HIV-infected children and adolescent on ART for at least 6 months and assessed its associated factors in the littoral region of Cameroon.

## Materials and methods

### i) Study design and population

A prospective population-based cohort study was conducted from November 2018 to October 2019 in the Littoral Region of Cameroon two years after the start of “*test and treat*” strategy in Cameroon. This region is divided into four departments (Wouri, Moungo, Nkam and Sanaga Maritime) and its capital is Douala an economic capital of the country. With an estimated average annual population growth rate of 5% the last 31 years and a current population of approximately 3,270,000 inhabitants, Douala is therefore the most populated city of Cameroon.

The Littoral Region of Cameroun concentrates about 20% (30,000) of people living with HIV (PLHIV) on ART in Cameroon. During the study period, about 2,200 children (aged less than 10 years old) and adolescent (aged 10 years to 19 years) on ART were followed up in two Accredited HIV Care Centers (AHCC) and 36 Decentralized HIV Care Unit (DHCU) mainly in Douala. One AHCC (Douala Laquintinie hospital-DLH) and six DHCU (Nkongsamba regional hospital-NRH, Nylon District hospital-NDH, Deido District hospital-DDH, Cité des palmiers District hospital-CDDH, Saint Albert Legrand catholic hospital-SALCH and Soboum medical Center-SMC) which concentrated about 1,150 (52.3%) children and adolescents on ART were selected according to the health facilities level and the number of patients followed up.

We used data sources from the previous studies [19] and assuming a prevalence of VF rate of 20% and the margin of error of 5%, it was determined that a minimal sample size of 246 patients would be needed and calculated using the Cochran’s formula [31]. Finally, we used a convenience sampling a non-probability sampling applicable in the study when the members of the population are convenient to sample as long as we were above the minimum size and had good power. For limit the selection and information biases, HIV-infected children and adolescents were enrolled consecutively and participation in the study was voluntary.

The survey’s target population was all HIV-infected children (0–9 years) and adolescents (10–19 years) on at least 6 months ART and regularly followed-up in the pediatric wards of the 7 seven selected health facilities.

At the inclusion, VL1 test was carried out and when VL1 was ≥ 1000 RNA copies/ml of blood plasma, an assessment of compliance was carried out by an educator and the 2^nd^ VL (VL2) was performed between 3 and 6 six months later according to national guidelines. VLS was defined as VL < 1000 RNA-copies/mL of blood plasma [32,33].

This study was conducted in accordance with ethics directives related to research on humans in Cameroon. The study received an ethical clearance from the Institutional Health Research Ethics Committee of the University of Douala (N°2437/CEI-UD/08/2020) and, administrative authorization was obtained from all health facilities and the Regional delegation of public health for the Littoral. Before enrollment and the administration of questionnaire, patients/tutors were informed on the purpose and process of the investigation (background, goals, methodology, study constraints, data confidentiality, and rights to opt out from the study). Adolescents were allowed to give their own written assent to participate in the study if they were judged by the study nurses to be able to give consent, and signed informed consent was obtained from the parents/guardians of children and adolescents in accordance with the Helsinki declaration and national guidelines. Participation was voluntary, anonymous and unpaid. Participation was voluntary, anonymous and without compensation.

### ii) Study questionnaire

They were closed-ended interview questions, including a single answer, and multiple choices questions. Data collection sheets were used to collect data on socio-demographic characteristics (year of birth, gender, place of residence, parents’ life status and residence family). The second part focused on i) clinical initiation status and, ii) therapeutic history (date on ART start and current ART regimen) were collected from registers and medical records. Administered questionnaire was conducted following a 1-week pre-test among 10 patients or parents/tutor per site to assess: i) understanding and acceptability of participants to the study and ii) to standardize and homogenize data collection in all sites. Interview questions were formulated so as not to influence participants in their answers.

### iii) Blood sampling and HIV-1 plasmatic RNA isolation using quantitative RT-PCR assay

In the follow up health facilities, 1,000 microL of plasma were separated from venous blood samples (4 ml) collected into EDTA (Ethylene diamine tretracetic acid) vacutainer tubes after centrifugation (10.000 rotations per minute for 10 minutes). Plasma was then transferred in the cryobox and stored at -80°C to the molecular biology laboratory of the Douala Laquintinie Hospital until use for the HIV-1 plasmatic RNA quantification.

HIV-1 RNA quantification was performed on plasma samples using the Abbott m2000 RealTime HIV-platform according to manufacturer recommendations (Abbott Molecular Inc. 1300 E. Touhy Ave. Des Plaines, IL 60018 200680–105; USA). We used 50 μl of extracted RNA and 50 μl of Master Mix for a total volume of 100 μl. The tests were carried out at the molecular biology laboratory at DLH. After plasma centrifugation at 5000g for 5 minutes, a protocol using 600 microL of plasma was used for RNA extraction with the Abbott m2000sp automate. Individual eluated RNA extraction was diluted at 1/2 (Master Mix for a total volume of 100 μl) was then deposited in the microplates and amplified with the Abbott m2000rt thermal cycler. The lower limit of detection of the assay is < 40 copies/mL of HIV-1 RNA and the upper limit is 10.000.000 copies/mL.

### iv) Statistical analysis

Data were presented as mean, median, interquartile range (*IQR; 25*%–*75*%) and percentage in tables and/or charts. Multivariate logistic regression analysis was used to identify the determinants of VF. The odd ratios (OR) were computed to appraise the strength of association between dependent and independent variables, accounting for the potential confounding factors. Significance was set at *p-values* less than 5%. All statically analyses were performed using statistical package for social sciences (SPSS) version 20.0 for Windows (SPSS. Inc. Chicago. U.S.A) and Stata software version 11 for Windows.

## Results

### Viral load success

**Fig 1:**
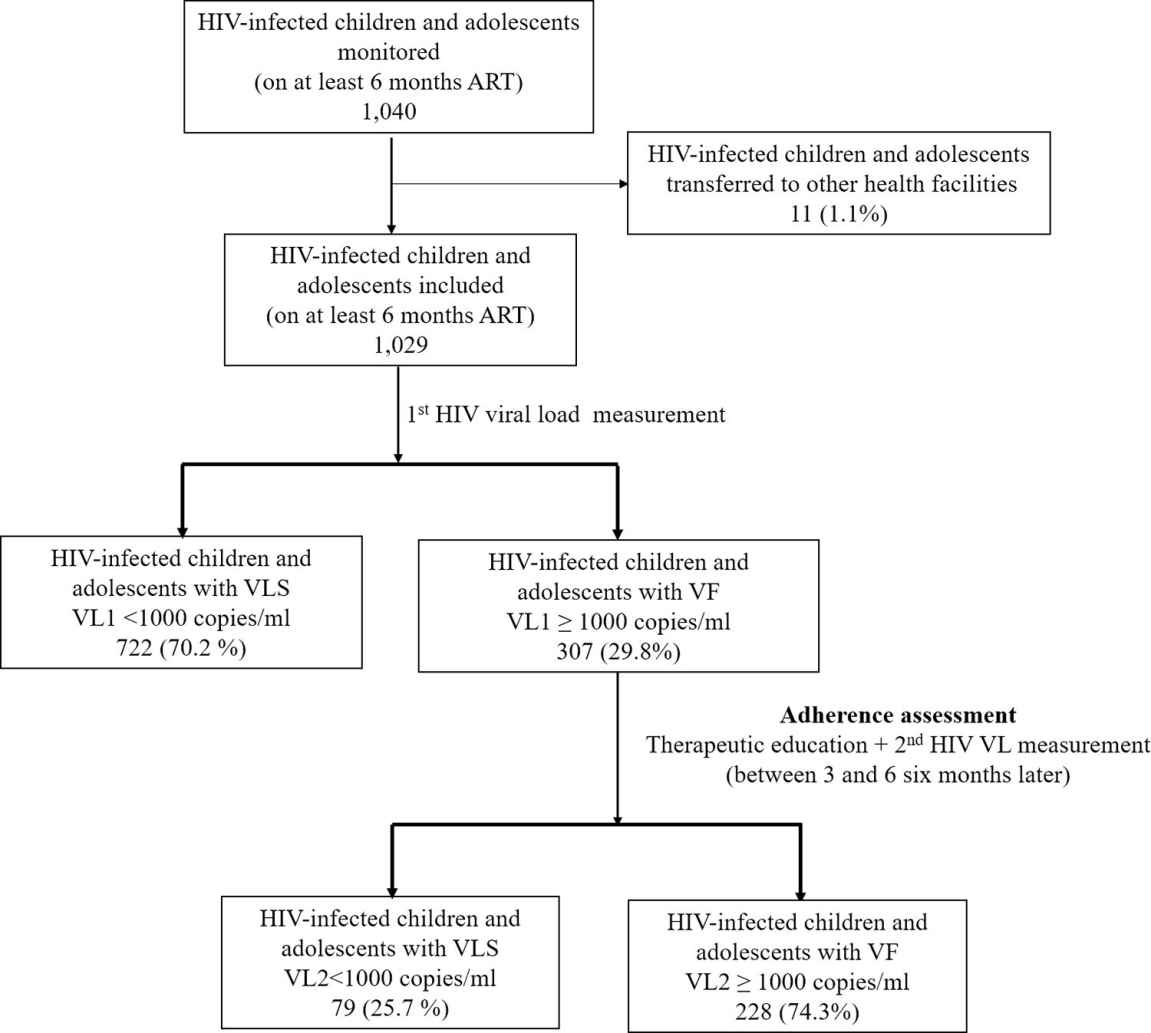
Flow chart of patients through the study according to the viral load monitoring. **Note.**; ART, antiretroviral treatment; VL, viral load; VLS, viral load suppression; VF, virologic failure.

At baseline, 1,040 children and adolescents monitored in the seven health facilities were invited to participate, of whom 11 (1.1%) were transferred to other health facilities during the study. Transfers were most often requested by parents/guardians after they have moved out of the city or to neighborhoods that are some distance away from the seven health facilities selected. Finally, a total of 1,029 patients were included in the seven health facilities, 427 (41.5%) in DLH, 62 (6.0%) in NRH, 167 (16.2%) in NDH, 89 (8.6%) in DDH, 130 (12.6%) in CDDH, 73 (7.1%) in SALCH and 81 (7.7%) in SMC (Fig 1). Most of HIV-infected children and adolescents (70.2%, 722/1029) on ART had a suppressed VL1 (VL1<1000 copies/ml) at the inclusion and among patients with a VLNS, 79 (25.7%) had suppressed their VL after therapeutic education. Ultimately, after the first VL measurement and the second VL performed after therapeutic education, the rate of VF is 22.2% for the whole HIV-infected children and adolescents population.

### Characteristics of participants in population survey and virologic failure

Children accounted for 393 (38.2%) of the patient population and 636 (61.8%) were adolescents (Table 1). The median age of HIV-infected children and adolescents was 11.7 years (IQR_25-75%_: 7.6–15.4; min-max: 7.0–19.6 years). Patients were mostly girls (52.5%) and 38.0% were orphans.

In total, 77.8% (801/1.029) had a suppressed VL after the first and the second test and among them, 40.1% (321/801) were children and 59.9% (480/801) were adolescents (Table 1).

**Table 1:** Patient’s characteristics visiting the health facilities by viral load status.

Variables	VF^$^ 228 (22.2)	VLS^£^ 801 (77.8)	Total 1,029	p-value	OR [CI 95%]^&^
**Age group, years**					
Children	72 (18.3)	321 (81.7)	393 (38.2)	0.020^≠^	1.45 [1.06–1.98]
[0–5]	27 (19.3)	113 (80.7)	140 (13.6)	Reference	
[5–10]	45 (17.8)	208 (82.2)	253 (24.6)	0.713	0.91 [0.53–1.54]
Adolescents	156 (24.5)	480 (75.5)	636 (61.8)	/	/
[10–15]	93 (27.1)	250 (72.9)	343 (33.3)	0.072	1.56 [0.96–2.52]
[15–20]	63 (21.5)	230 (78.5)	293 (28.5)	0.595	1.15 [0.69–1.89]
**Gender**					
Female	121 (22.4)	419 (77.6)	540 (52.5)	0.839	0.97 [0.72–1.30]
Male	107 (21.9)	382 (78.1)	489 (47.5)		
**Parents’ life status**					
Both alive	120 (18.8)	518 (81.2)	638 (62.0)	Reference	
Mother died	49 (26.2)	138 (73.8)	187 (18.2)	0.028	1.53 [1.04–2.24]
Father died	30 (28.0)	77 (72.0)	107 (10.4)	0.029	1.68 [1.05–2.68]
Both parents died	29 (29.9)	68 (70.1)	97 (9.4)	0.012	1.84 [1.14–2.97]
**Residence family**					
With Parents	104 (18.5)	458 (81.5)	562 (54.6)	Reference	
With grand parents	24 (24.7)	73 (75.3)	97 (9.4)	0.153	1.45 [0.87–2.40]
With mother	31 (27.2)	83 (72.8)	114 (11.1)	0.036	1.65 [1.03–2.62]
With father	24 (26.7)	66 (73.3)	90 (8.7)	0.072	1.60 [0.96–2.68]
With other family member*	45 (27.1)	121 (72.9)	166 (16.1)	0.016	1.64 [1.09–2.45]
**Heath facility**					
Accredited HIV Care Center	71 (16.6)	356 (83.4)	427 (41.5)	<0.001	1.76 [1.29–2.41]
Decentralized HIV Care Unit	157 (26.1)	445 (73.9)	602 (58.5)		
**Duration on ART, months**					
Median [_IQR25-75_]^μ^	44.9 [19.2; 83.4]	48.7 [24.7; 95.2]	48.2 [23.7; 92.8]	0.077	
≤24	68 (25.7)	197 (74.3)	265 (25.8)	0.183	1.26 [0.89–1.79]
[24–49]	49 (19.9)	197 (80.1)	246 (23.9)	0.632	0.91 [0.62–1.32]
≥49	111 (21.4)	407 (78.6)	518 (50.3)	Reference	
**ART regimen exposure since the initiation**					
NRTI^α^ + PI/r^π^ combination	15 (17.6)	70 (82.4)	85 (8.3)	Reference	
NRTI+ NNRTI^€^ combination	165 (22.1)	583 (77.9)	748 (72.7)	0.350	1.32 [0.73–2.36]
Switched from NNRTI to PI/r	48 (24.5)	148 (75.5)	196 (19.0)	0.208	1.51 [0.79–2.88]
**Number of ART molecules received since initiation**					
3	87 (22.4)	301 (77.6)	388 (37.7)	Reference	
4–6	127 (21.9)	454 (78.1)	581 (56.5)	0.836	0.96 [0.71–1.31]
≥7	14 (23.3)	46 (76.7)	60 (5.8)	0.875	1.05 [0.55–2.00]

***Note*.** Data are number and/or proportion (%), otherwise indicated. ^**$**^, virologic failure^**£**^, Viral load suppression; *, aunt, uncle, brother or sister; ^μ^, interquartile range 25–75%; ^α^, Nucleoside reverse transcriptase inhibitor; ^π^, Protease inhibitor boosted with ritonavir; ^€^, Non-nucleoside reverse transcriptase inhibitor; ^**&**^, Odd Ratio [95% confidence range]; Comparison had been done between VLNS group and VLS group for the variable indicated; ^≠^, Comparison between children group and adolescents.

Adolescents were significantly more likely than children to have VF (24.5% *vs* 18.3%, OR = 1.45, 95%CI: 1.06–1.98, p *=* 0.020). The number of patients with VF was significantly higher among maternal orphans (26.2% *vs* 18.8%, OR = 1.53, 95%CI: 1.04–2.24, p = 0.028), father orphans (28.0% *vs* 18.8%, OR = 1.68, 95%CI: 1.05–2.68, p *=* 0.029) or orphans of both parents (29.9% *vs* 18.8%, OR = 1.84, 95%CI: 1.14–2.97, p = 0.012) compared to those who had both parents. VF was higher in patients living with their mother (27.2% versus 18.5%, OR = 1.65, 95% CI: 1.03–2.62, p = 0.036) and with other family members (27.1% vs 18.8%, OR = 1.64, 95% CI: 1.09–2.45, p = 0.016).

Although no statistically significant difference was observed when comparing the medians, the mean duration of exposure to ART was significantly lower in patients with VF compared to VLS group (55.7 months *vs* 55.7 months for VLS group, p = 0.043) (Table 1). The number of patients with VFwas significantly higher in DHCU (26.1% vs 16.6%, OR = 1.76, 95%CI: 1.29–2.41, p<0.001) compared to AHCC. No association was found between VL status and ART combination or number of ART molecules received since the initiation of ART.

### Virologic failure and associated factors

To identify the demographic and therapeutic risk factors that associated independently with VLNS in the population, multivariate analyses was done using age group, heath facility, parents’ life status and residence family. As shown in Table 2, adolescents attending the DHCU were independently associated with VF.

**Table 2:** Multivariate analysis of factors associated with virologic failure.

Variables	Overall population		Children’s group		Adolescents’ group	
	OR [95% CI]^&^	p value	OR [95% CI]^&^	p value	OR [95% CI]^&^	p value
**Adolescents ([10–20 [years)**	1.39 [0.99–1.93]	0.050	/	/	/	/
**DHCU** ^£^	1.87 [1.36–2.57]	<0.001	1.64 [0.91–2.98]	0.099	1.92 [1.31–2.83]	0.001
**Father died**	1.29 [0.55–3.01]	0.551	0.71 [0.15–3.36]	0.670	1.95 [0.64–5.90]	0.233
**Mother died**	1.36 [0.64–2.89]	0.419	1.76 [0.32–9.73]	0.516	1.55 [0.64–3.75]	0.327
**Both parents died**	1.51 [0.70–3.23]	0.291	1.22 [0.14–10.34]	0.851	1.71 [0.71–4.07]	0.225
**Residence with Grand-parents**	1.05 [0.49–2.27]	0.892	0.76 [0.15–3.88]	0.745	1.09 [0.43–2.77]	0.852
**Residence with mother**	1.26 [0.54–2.90]	0.589	1.79 [0.55–5.80]	0.329	0.95 [0.29–3.05]	0.935
**Residence with father**	1.20 [0.49–2.95]	0.686	0.09 [0.007–1.32]	0.080	1.88 [0.66–5.30]	0.231
**Residence with other family’s members**	1.15 [0.57–2.28]	0.689	0.47 [0.08–2.62]	0.394	1.35 [0.60–3.04]	0.465

***Note*.** ^**£**^, Decentralized HIV care units: ^**&**^, Odd Ratio [95% confidence range]; Comparison had been done between virologic failure group and viral load suppression group for the variable indicated.

## Discussion

Virological surveillance is the main method used to assess the effectiveness of ART in PLWH. VL monitoring can therefore help to detect treatment failure and determine whether the patient should be switched to another treatment regimen. This study describes the virological response and factors associated with VF on 1,029 HIV-infected children and adolescents (393 children and 636 adolescents) routinely followed in seven health facilities in the Littoral region of Cameroon. This is one of the limited studies assessing VLNS in a large cohort involving more than a thousand HIV-infected children and adolescents in Central Africa and particularly in Cameroun.

Among HIV-infected children and adolescents on ART for at least 6 months, 70.2% had undetectable VL at their first VL assessment, whereas the remaining 29.8% had received adherence counselling sessions before the second VL test. The intervention with the therapeutic education increased the VLS from 70.2% to 77.8% in the entire pediatric population after the two VL measurements separated by 3 to 6 months, thus reducing the number of patients with VLNS at 22.7%. This approach used in our study in determining virological response to ART punctuated by therapeutic education could explain the difference observed with several other studies conducted in the same area [24] and in other regions of Cameroon [20–22,34], in Africa [14,26,27,29] and in Europe [35–37]. In these studies, VLNS rates vary between 20% and 64% after a single VL test. The positive contribution of therapeutic education to VLS was also observed in a previous study conducted in one of the AHCC included in this study, the DLH in 2016, where the authors reported that virological response appears to be better in children/adolescents who acquire skills more quickly through therapeutic education and therefore require fewer learning sessions to adapt [38].

Children had a better virological response than adolescents. A similar result was reported in a study conducted in Cameroon among patients on ART recruited in the Center, South and East Regions of Cameroon [22]. Similarly, two other studies conducted in Ethiopia reported a higher risk of VF among adolescents (age 10–15 years) on ART compared to those aged 6–9 years [28,39]. Adherence to ART in children depends on parents/guardians who are responsible for drugs procurement and their administration to children. This could explain the difference reported in virological response with adolescents who are in a period of biological, physiological and psychological growth with increasing acquisition of social autonomy. Adolescents are involved in the drugs procurement and self-administration, which increases the risk of poor adherence to treatment [40]. Thus, ART observance among adolescents should be continuously boosted by therapeutic educators throughout the antiretroviral drugs intake.

In this study and in another among HIV-infected children in Senegal, the VF rate was higher among patients routinely followed up in the DHCU compared to those followed up in the AHCC [29]. In Cameroon, AHCCs are 1st and 2nd category hospitals in the health pyramid in Cameroon, while DHCU are district hospitals or peripheral medical centers. The latter health centers are characterized by a weak technical platform, limited logistics and health personnel often with little or no training, which would explain the low rate of viral success observed in the DHCU. The reference laboratories that carry out viral load tests are only installed in the AHCCs. Then all the DHCU collect blood samples and send them to reference laboratories for viral load tests. It would be wise to deploy the logistical means for the rapid and efficient transport of samples or to accelerate the point of care effort which is underway in certain DHCUs for an early and efficient diagnosis of VF in HIV-infected children and adolescents.

VLS was lower among adolescent orphans and who did not live with their parents. A similar trend was observed in a previous study in Ethiopia where the death of both parents was a risk factor for VF among pediatric population [40]. Another studies conducted in Europe and in Africa reported that children whose ART care was not on the direct responsibility of both parents had a higher risk of treatment failure compared to those living with neither or one of the parents [36,37,41]. In countries with limited resources, orphan status has a significant negative impact on the social status of children and adolescents in care situations in terms of financial, material and emotional support. These various factors, together with the stress of the disease, can have a significant impact on orphans’ adherence to ART [42].

The ART duration was not associated with VF in this study, whereas several other studies conducted in pediatric populations on ART showed that ART duration less than 48 months [27,43] and ART use for more than 1 year or more than 3 years [26] was a risk factor for VF. This could be explained by the fact that the virological data of the participants in our study were not obtained from the time of ART initiation to determine the time of VF onset in children and adolescents on ART.

This could be explained by the fact that the virological data of the participants in our study were not obtained from the time of ART initiation to determine the time of VF onset in children and adolescents on ART.

## Conclusion

The current study shows that VF remains a major challenge for HIV-infected children and adolescents on ART and particularly among adolescents. HIV-infected adolescents and patients followed-up in a DHCU are more at risk of VF and therapeutic education improves the virological response. A specific intervention targeting adolescents in particular, patients followed in the DHCUs and orphans because of their precarious social status, would be necessary.

## Abbreviations

AHCC: accredited HIV care center
ART: antiretroviral treatment
CDDH: Cité des palmiers District hospital
CI95%: 95% Confidence interval
DDH: Deido District hospital
DHCU: Decentralized HIV care units
DLH: Douala Laquintinie hospital
EDTA: Ethylene diamine tretracetic acid
HIV: Human immunodeficiency virus
HIVDR: HIV drug resistance
IQR_25-75%_: Interquartille 25–75% range
NDH: Nylon District hospital
NRH: Nkongsamba regional hospital
NRTI: Nucleoside reverse transcriptase inhibitor
NNRTI: Non-Nucleoside reverse transcriptase inhibitor
NRH: Nkongsamba regional hospital
OR: Odd ratio
PI/r: Protease inhibitor boosted with ritonavir
PLHIV: people living with HIV
RNA: Ribonucleic acid
SALCH: Saint Albert Legrand catholic hospital
SD: Standard deviation
SMC: Soboum medical Center
VF: Virologic failure
VL: Viral load
VLS: Viral load suppression
WHO: World Health Organization
